# Description of an automatic copulation induction system used to establish a free-mating laboratory colony of *Nyssorhynchus deaneorum* from Brazil

**DOI:** 10.1590/0074-02760200070

**Published:** 2020-07-08

**Authors:** Maisa da Silva Araujo, Najara Akira Costa dos Santos, Alice Oliveira Andrade, Raphael Brum Castro, Alessandra da Silva Bastos, Fábio Resadore, Luiz Hidelbrando Pereira-da-Silva, Jansen Fernandes Medeiros

**Affiliations:** 1 Fundação Oswaldo Cruz-Fiocruz, Laboratório de Entomologia, Porto Velho, RO, Brasil; 2 Universidade Federal de Rondônia, Programa de Pós-Graduação em Biologia Experimental, Porto Velho, RO, Brasil; 3 Fundação Oswaldo Cruz-Fiocruz, Instituto Nacional de Epidemiologia da Amazônia Ocidental, Porto Velho, RO, Brasil; 4 Fundação Oswaldo Cruz-Fiocruz, Laboratório Epidemiologia Genética, Porto Velho, RO, Brasil; 5 Fundação Oswaldo Cruz-Fiocruz, Instituto Oswaldo Cruz, Programa de Pós-Graduação em Biologia Parasitária, Rio de Janeiro, RJ, Brasil

**Keywords:** Nyssorhynchus deaneorum, copulation induction, colony establishment, malaria vector

## Abstract

**BACKGROUND:**

*Nyssorhynchus deaneorum* is a potential malaria vector because it has been shown to be competent to transmit *Plasmodium vivax* and *Plasmodium falciparum*, and because it exhibits antropophilic and endophilic behaviors in some regions of the Amazon. This profile makes *Ny. deaneorum* a useful mosquito for experiments that model *Plasmodium-*vector interactions in the Amazon.

**OBJECTIVE:**

Herein we describe how a free-mating colony of *Ny. deaneorum* has been established using an automated light stimulation system.

**METHODS:**

Mosquitoes were captured in São Francisco do Guaporé, Rondônia. The F1 generation was reared until adult emergence at which point copulation was induced using an automatic copulation induction system (ACIS).

**FINDINGS:**

After four generations, natural mating and oviposition began to occur without light stimulation. The number of pupae and adult mosquitoes increased from the F_5_ to F_10_ generations. The new *Ny. deaneorum* colony exhibited susceptibility to *P. vivax.*

**MAIN CONCLUSIONS:**

Automated light stimulation is an effective method for establishing an *Ny. deaneorum* colony under laboratory conditions as it produces enough adults to create a stenogamic colony. The establishment of a stable, *P. vivax*-susceptible colony of *Ny. deaneorum* makes it possible to model parasite-vector interactions and to test novel drug therapies that target parasite development in mosquitoes.

*Nyssorhynchus darlingi* (Root) is the main vector of malaria in the Amazon Region; however, other anopheline mosquitoes play an important role in the transmission of *Plasmodium*.^1^ ^,^ ^2^ In particular, mosquitoes from the Albitarsis Group have a significant epidemiological impact at regional and local levels. *Nyssorhynchus albitarsis* s. l. is widely distributed (from northern Guatemala to northern Argentina), and exhibits morphological and behavioral variation; the group has been divided into separate species on the basis of morphological and genetic differences.^3^ ^,^ ^4^

The current literature recognises five species of *Ny. albitarsis* s. l.: *Nyssorhynchus albitarsis* (Lynch-Arribálzaga), *Nyssorhynchus marajoara* (Galvão & Damasceno), *Nyssorhynchus deaneorum* (Rosa-Freitas), *Nyssorhynchus oryzalimnetes* (Wilkerson & Motoki) (*Nyssorhynchus albitarsis* B), *Nyssorhynchus janconnae* (Wilkerson & Sallum) (*Nyssorhynchus albitarsis* E). The group also includes four unnamed species: *Nyssorhynchus albitarsis* F, *Nyssorhynchus albitarsis* G, *Nyssorhynchus albitarsis* I, and a mitochondrial lineage, *Nyssorhynchus albitarsis* H.^5^ ^,^ ^6^ Three of these species contribute to malaria transmission in certain regions of the Amazon: *Ny. marajoara*, *Ny. janconnae* and *Ny. deaneorum*.^2^ ^,^ ^7^ ^,^ ^8^ ^,^ ^9^ ^,^ ^10^

*Nyssorhynchus deaneorum* is morphologically distinct and has a unique isoenzyme profile; it was originally described on the basis of specimens captured in Rondônia and Acre.^11^ The current geographic distribution of *Ny. deaneourum* includes west, mid-west and southern Brazil, Bolivia and northern Argentina.^4^ ^,^ ^5^ ^,^ ^12^ ^,^ ^13^ ^,^ ^14^

In general, *Ny. deaneorum* has been associated with low to medium human population and has been found primarily in rural areas where malaria is more prevalent.^15^ Once *Ny. deaneorum* has been shown to be both naturally and experimentally susceptible to *Plasmodium vivax* and *Plasmodium falciparum*, it will be important to evaluate its vectorial competence given that *Ny. deaneorum* exhibits antropophilic and endophilic behaviors, and occurs in high density in some regions of Rondônia and Acre.^7^ ^,^ ^8^ ^,^ ^9^ ^,^ ^16^ ^,^ ^17^ ^,^ ^18^ In an ecological niche model study, the distribution of *Ny. deaneorum* was associated with *P. falciparum*, which confirms its role in malaria transmission.^15^ Furthermore, *Ny. deaneorum* is considered a climate generalist capable of achieving significant geographic expansion in the advent of changes to the climate and biome; this capacity is likely to elevate the importance of *Ny. deaneorum* in malaria transmission in the future.^6^ Given the potential of *Ny. deaneorum* to act as a primary vector in some regions, its ecology, behavior, biology, genomics and vector-parasite interaction will need to undergo further study in order to properly assess this species role in malaria transmission.^6^

In 1988, a colony of *Ny. deaneorum* was started in Costa Marques-RO/Brazil using the forced mating technique. The colony was maintained for more than 25 generations but was not permanently established.^19^ This colony was used to study taxonomy and susceptibility to *P. vivax* and *P. falciparum*, which made it possible to assess *Ny. deaneorum* vectorial competence.^8^ ^,^ ^9^ Without large scale routine production of *Ny. deaneorum*, experimental trials cannot be performed. Until now, studies have been limited to mosquitoes captured in the field, which means that vector capacity assessments have been restricted to studies of the natural susceptibility of *Ny. deaneorum* to *Plasmodium* parasites.^7^ ^,^ ^20^

Herein, we describe the establishment of a free-mating colony of *Ny. deaneorum* that was achieved by automating a natural copulation induction technique initially developed to breed *Ny. darlingi*.^21^ This automated system provides an effective, practical and rapid means of establishing and mass rearing *Nyssorhynchus* species in an insectary.

## MATERIALS AND METHODS

*Field collections and F1 adult production* - A colony of *Ny. deaneorum* was initiated using 399 adult females. Mosquitoes were collected exclusively outdoors and close to houses using BG-Malaria traps^21^ and human landing catches in São Francisco do Guaporé, Rondônia State (8°39’8. 874’ S / 63°56’8.106 W), 604 km from Porto Velho, Rondônia State. Collections were made at dusk (from 6:00 p.m. to 9:00 p.m.) for two consecutive evenings during July of 2018. Captured females were fed using the authors’ blood samples. Approximately 2 mL of this blood was placed on the exterior base of inverted plastic cups (80 mL) and a polytetrafluoroethylene membrane was stretched over the blood to seal it in place. Cups were turned right-side up and filled with boiled water to keep the blood warm (~37ºC), and then held for 15 min over netting cages containing 30-50 mosquitoes. A 10% sacarose solution was offered to females after blood feeding and the females were transported to the Fiocruz/RO Entomology Laboratory in Porto Velho, Brazil.

Once in the laboratory, engorged females were maintained in the insectary at 26 ± 1ºC and 70 ± 10% relative humidity under a 12:12 h light-dark cycle. After 72 hours, field-collected females were anesthetised and identified using Consoli and Lourenço-de-Oliveira.^22^ Following identification, one wing was removed from *Ny. deaneorum* mosquitoes in order to induce oviposition and females were placed in a Petri dish that was lined with filter paper moistened with distilled water (15 females/plate). Over the course of three days, females were transferred to clean moistened plates, and then the eggs were washed in sodium hypochlorite solution (0.1%) to stimulate hatching and then placed in plastic pans (30.3 x 22.1 x 7.5 cm) containing 1 liter of distilled water.

After five days, larvae were placed in clear plastic pans (30.3 x 22.1 x 7.5 cm). Each pan contained 1 liter of distilled water at a density of 200 larvae. Larvae were maintained by changing the water three times a week and adding 5 mL of 1% yeast solution for nutritional supplementation. Larvae were fed on finely ground Tetramin Tropical Fish Food^®^ sprinkled on the surface of the water once a day during the first and second instars (size of larvae food: 63 μm and 125 μm, respectively), and three times a day during the third and fourth in stars (size of larvae food: 250 μm). The quantity of food was spread sufficiently to avoid water turbidity. Pupae were transferred to plastic containers (500 mL) filled with 250 mL of distilled water (500 pupae/container) and containers were placed in screen cages (61 x 61 x 61 cm). Emerged adults were fed a 15% honey-water solution, *ad libitum*.

**Fig. 1:**
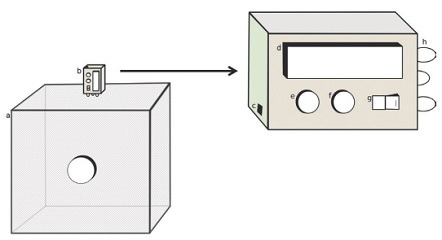
scheme of automatic copulation induction system (ACIS) placed on a cage to induce copulation. (a) Cage of mosquitoes (61 x 61 x 61 cm); (b) automatic copulation induction system (ACIS, 12 x 5.5 x 7cm); (c) power supply input; (d) LCD display module; (e) push buttons to start program cycle; (f) push buttons to start program cycle; (g) on/off button; (h) three white copulation induction LEDs.

*Induced copulation by automatic copulation induction system (ACIS)* - The ACIS (Fig. 1) was developed on an Arduino system using the Arduino Nano V3 board. The automatisation program was written in C and compiled on the board’s chip. The DS3231 real time clock (RTC) module was used for time control in the automated software. A 16x2 LCD display module was used to monitor system status. The light emissions used to induce copulation were generated by three white 5 mm LEDs (3.2V; 6,000K; 18,000MCD). Push buttons were used to set the automated start/stop time of light cycles and to manually start and stop the automated process. The Arduino board was powered with a 9V power supply plugged directly into an outlet. The software was programmed to let the system run through the same induced copulation cycle daily without manual assistance.

The system was programmed as follows to induce *Ny. deaneorum* copulation: (i) at 6:00 p.m. the temperature of the insectary was reduced to 24 ± 1ºC and the light was turned off; (ii) at 6:30 p.m. the light beam cycles began (4 cycles of 10 min on / 10 min off); (iii) at approximately 7:50 p.m. the light beam cycles ended, the temperature was returned to 26 ± 1ºC and the 12:12 h light/dark cycle resumed.

Approximately 600-2,000 three to five-day-old *Ny. deaneorum* males and females (ratio 1:1) were placed in a cage to begin copulation induction. Mosquitoes were provided with honey-water solution 30 min before the temperature was lowered, and copulation induction was carried out for seven consecutive evenings. During the first six nights of induction, after the final light cycle, females were fed with chicken blood for 15 min (Ethical Committees on Animal Use of Fiocruz Rondônia - CEUA - approval 2014/05). Dead males and females were removed from cages each day before induction; their numbers were recorded and used to estimate the survival rate of adults for all periods of copulation induction. During the seven days of light stimulation, the number of copulations were recorded as the number of mating pairs who met in flight and fell to the cage floor. Results are presented here as the total number of copulations per day per light cycle. These direct observations confirmed that mosquito copulation was being effectively induced by the ACIS.

Nine days after the initial induction, oviposition was induced by cutting one wing of each female, as described above. Copulation induction was performed until natural copulation was observed.

*Subsequent generations and colony maintenance* - Natural mating behavior was assessed by comparing caged egg laying (induced oviposition) in the copulation-induced system versus non-stimulated controls. In order to evaluate natural oviposition, three days after the first blood meal small black cups of distilled water were introduced to the cages to serve as an oviposition substrate.

After natural mating and oviposition was achieved, a simple standard protocol was established to rear and maintain subsequent generations: Adults were fed a 15% honey-water solution in saturated cotton. Three to five-day old mosquitoes (1,000-2,000 mosquitoes per cage) were provided with honey-water solution for approximately nine hours and fed on chicken blood for 15 min (CEUA 2014/05) over two consecutive days. After 72 hours, small black cups with distilled water were introduced as described above to encourage natural oviposition. On the third day, the eggs were washed as described above, and on the fifth day larvae were transferred to new pans (200 larvae/pan). Larvae rearing followed the same procedure used to obtain the F_1_ adult mosquitoes. Standardised oviposition and larvae rearing have been used to rear *Ny. deaneorum* up to the F_10_ generation.

Molecular verification of wild type, F_1_, F_2_, F_3_ and F_4_ *Ny. deaneorum* adults was confirmed using mitochondrial cytochrome c oxidase I (COI mtDNA) gene sequences.^23^ The purified fragments were sent to the Sequence Platform of Fiocruz Minas Gerais/Brazil.

*Plasmodium vivax infection* - In order to assess the susceptibility of our *Ny. deaneorum* colony to *P. vivax*, membrane feeding assays (MFA) were performed on different generations. Trials were performed using volunteers, 18 years and older, who were *P. vivax* symptomatic and infected with gametocytes as diagnosed by microscopy (Protocol approved by the Brazilian National Ethics Committee Board - CEP 2.641.046). Approximately 50-80 three to five-day old female mosquitoes were fed on infected blood via MFA for 30 min. After feeding, fully fed females were kept under insectary conditions and maintained with 15% honey-water solution daily, until dissection. Midguts were dissected seven days after blood feeding and oocysts were stained with 0.2% mercurochrome and counted under x10 magnification. Salivary glands were dissected 14 days after feeding and the number of sporozoites were counted under x40 magnification using a Neubauer chamber.

## RESULTS

The COI mtDNA sequences from wild type, F_1_, F_2_, F_3_ and F_4_ generations exhibited the highest identity (99-100%) with GenBank sequences from Mato Grosso, Mato Grosso do Sul, Rondônia and Acre, Brazil (KJ492739, KJ492762, JQ615333, MH844252).^5^ ^,^ ^24^

During copulation induction by light stimulation, the number of copulations per day increased from day one to day four; copulations were highest on day four and then decreased (as shown in Table I). The highest number of copulations occurred during the first and second light cycles (Table I). Flight activity was also highest at these times because the mosquitoes formed pseudo swarms composed primarily of males. Copulations decreased in number from the first to last light cycle. More copulations occurred in cages that held higher numbers of mosquitoes.

**TABLE I:** Number of copulations recorded for light induction period for *Nyssorhynchus deaneorum* during seven days and for light cycles

Days of induced	Number of copulations per light cycle				Total (Mean ± SD)
	1st cycle	2nd cycle	3rd cycle	4th cycle		
1	12	11	8	3	34 (9 ± 4.2)
2	56	22	11	9	98 (25 ± 21.7)
3	32	27	17	11	86 (21 ± 9.5)
4	64	26	15	10	114 (29 ± 24.2)
5	27	31	21	12	90 (23 ± 8.4)
6	20	30	18	8	76 (19 ± 9.0)
7	18	25	12	5	60 (15 ± 8.5)
Total (Mean ± SD)	229 (33 ± 19.6)	172(25 ± 6.6)	101 (14 ± 4.4)	57 (8 ± 3.2)	

SD: standard deviation

**Fig. 2:**
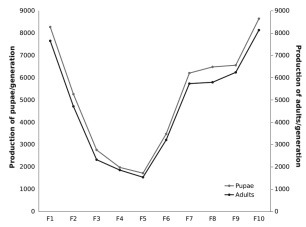
number of *Nyssorhynchus deaneorum* pupae and emerged adults for generations F_1_-F_10_.

Table II shows the number of mosquitoes used in copulation induction, the survival rates of males and females and the percentage of oviposition. Adult survival rates during copulation induction increased slightly from F_1_ to F_10_ but males seemed less robust than females. Consequently, the percentage of oviposition increased with successive generations. The production of pupae and adults decreased from F_1_ to F_5_; however, after the F_5_ generation these numbers began to increase, rising from 1,714 pupae and 1,531 adult mosquitoes in the F_5_ generation to 8,643 pupae and 8,131 adult mosquitoes in the F_10_ generation (Fig. 2). Overall larvae and pupae mortality were low in all generations and this allowed the colony to perpetuate. In general, the emergence rate was higher following the F_5_ generation and ranged from 84.4% to 94.1%, with the lowest rate occurring in the F_8_ generation. Development time from one generation to the next was approximately three weeks. After four generations, natural mating and oviposition was observed without light stimulation from the ACIS.

**TABLE II:** Number of mosquitoes submitted to induction, mosquito survival rates after induction and percentage of oviposition

Generation	Number of mosquitoes per induction (1:1)	No. of cages	Survival % male/female	Total number of ovipositions (%)
Wild type				302 (76.0)
F1	6,186	3	34.6/34.8	281 (26.1)
F2	4,418	3	25.3/32.6	192 (26.2)
F3	2,582	2	32.6/43.1	164 (29.5)
F4	1,907	2	34.3/36.2	126 (36.5)
F5*	1,360	2	39.0/49.0	125 (37.5)
F6	3,382	2	31.0/39.0	232 (35.2)
F7	4,978	2	43.4/39.8	356 (36.0)
F8	5,432	2	35.8/37.0	437 (43.5)
F9	5,096	2	50.1/52.8	675 (43.3)
F10	7,800	4	49.5/52.3	1085 (53.2)

*: natural copulations and oviposition were registered without induction.

Samples from the F_2_, F_3_, F_6_ and F_8_ generations were submitted to MFA to test for *P. vivax* susceptibility. *P. vivax* infection rates ranged from 66.7% to 100% [mean ± standard deviation (SD) = 92.6% ± 11.4%], the number of oocysts per mosquito ranged from 1 to 112 (mean ± SD = 27.2 ± 35.4), and the number of sporozoites per mosquito ranged from 125 to 38,171 (mean ± SD = 3,025 ± 7,147).

## DISCUSSION

*Ny. deaneorum* is a useful mosquito to maintain under laboratory conditions because it can be used as an experimental model for studying *Plasmodium*-vector interactions. Klein et al.^8^ demonstrated that reared F_1_ of *Ny. deaneorum* are susceptible to *P. vivax* and exhibit *P. vivax* infection rates and number of sporozoite comparable to those of *Ny. darlingi*, a primary malaria vector throughout the Amazon Basin. Furthermore, in certain regions of Rondônia, such as Costa Marques and São Francisco do Guaporé, *Ny. deaneorum* density increases as *Ny. darlingi* density decreases during the early dry season (May to August).^(8,unpublished observations)^ Moreover, a study modeling future changes to the climate and biome predicts that *Ny. darlingi* may decrease in significance while some species of the Albitarsis Complex, including *Ny. deaneorum*, may play a role as either primary or secondary vectors of *P. falciparum* in South America.^6^

Currently in Brazil, only *Ny. darlingi* and *Nyssorhynchus aquasalis* colonies have been maintained under laboratory conditions for the purpose of experimental study.^21^ ^,^ ^25^ In general, South American mosquito vectors have proven difficult to maintain in the laboratory; the principal difficulty has been inducing free copulation in the confined space of a laboratory cage.^25^ A colony of *Ny. aquasalis* was successfully established by the use of forced mating,^26^ a technique that forces copulation after the male head has been removed.^27^ However, this technique failed when it was applied to *Ny. darlingi*.^28^ ^,^ ^29^

The first *Ny*. *deaneorum* colony was established in Costa Marques, Rondônia, Brazil, and maintained for 25 generations using the forced mating technique.^19^ However, it is difficult to breed and maintain a mass colony for long periods of time using this method because it is labor-intensive, time-consuming and impractical. In our attempts to colonise *Ny*. *deaneorum* by forced mating, oviposition was low, and this decreased adult production in every subsequent generation (data not shown).

An alternative technique for inducing copulation under laboratory conditions is the application of light stimulation in combination with temperature change. This technique was developed by Villarreal-Treviño et al.^30^ for establishing an *Anopheles pseudopunctipennis* colony, and it has also been used to establish *Ny. darlingi* colonies.^2^ ^,^ ^21^ ^,^ ^29^ We used this technique to establish *Ny. deaneorum* colony, but we achieved this using an automated system (ACIS) to control light stimulation and temperature change. This automated system proved to be a very efficient and easy way to establish an anopheline colony. Light stimulation was conducted for four generations and became unnecessary after the F_5_ generation.

During the seven days induction period, the number of copulations induced by the automated system were comparable to the number of copulations reported by Villarreal-Treviño et al.^29^ Copulations increased from day one to day four and then decreased; this suggests that the next attempt to colonise an important vector could be achieved by using light beam stimulation for as little as five days per generation. Colonisation of *An. pseudopunctipennis* was achieved with 10 days of light beam stimulation for the F_1_ to F_4_ generations; induction was conducted for five days in subsequent generations, but this had no effect on the percentage of insemination.^30^

Decreased copulation could be the result of a progressive decrease in mosquito density. Survival rates decreased over the course of induction, and, in general, copulation was more frequent in cages with more mosquitoes. Moreover, mortality was more common in males than females (Table II). Given that *Ny. deaneorum* is eurygamic, like the majority of anopheline species,^31^ the density of males is important because swarms are composed almost entirely of males and copulation may be occurring primarily when females enter the swarm.^32^ Males were more active during light-induced mating. They formed pseudo swarms at the beginning of each light cycle, and they were especially active during the first cycle which is when the greatest number of copulations occurred (Table I).

The ACIS proved to be an effective way to establish an *Ny. deaneorum* colony under laboratory conditions: successful copulation, oviposition and larval development were observed, and enough adults were produced to propagate successive generations. The success of *Ny. deaneorum* colonisation by ACIS was confirmed when the production of pupae and adults increased after the F_5_ generation (Fig. 1). This increase is probably the result of adaptation to the laboratory environment. After the F_5_ generation it was no longer necessary to use the ACIS because mating and oviposition were naturally occurring, which indicates the development and selection of a stenogamic colony.

The first laboratory colony of *Ny. deaneorum* in Brazil was established using the forced mating technique, but the process of adaptation was not described.^19^ However, the number of generations needed to establish autonomous colonies has been documented with respect to certain species and population origins.^29^ ^,^ ^30^ *Ny. aquasalis* requires the smallest number of generations to develop an autonomous colony and has been established by the F_2_ generation;^26^ free mating in *Ny. albitarsis* and *Ny. darlingi* from Brazil has been achieved in six generations,^21^ ^,^ ^33^ while *Ny. darlingi* colonies from Peru required nine generations to become established as free-mating populations.^28^ ^,^ ^29^

Establishing a stable mosquito colony under laboratory conditions makes it possible to conduct standardised experiments that elucidate several important aspects of mosquito biology. For example, experimental infection can be used to measure *Plasmodium* infectivity in mosquitoes. Our data for *Ny. deaneorum* susceptibility to *P. vivax* indicates that our colony could be used as a model for studying *Plasmodium*-vector interactions in the Amazon and thus be used to test novel drug therapies that target parasite development in mosquitoes.

The light stimulation technique can be effectively automated to facilitate the creation of new mosquito colonies under laboratory conditions and automation is an efficient alternative to the labor-intensive method of manual light stimulation.
